# Finite Element Analysis of Film Stack Architecture for Complementary Metal-Oxide–Semiconductor Image Sensors

**DOI:** 10.3390/s17051004

**Published:** 2017-05-02

**Authors:** Kuo-Tsai Wu, Sheng-Jye Hwang, Huei-Huang Lee

**Affiliations:** 1 Department of Mechanical Engineering, National Cheng Kung University, No. 1, University Road, Tainan 701, Taiwan; n18991194@mail.ncku.edu.tw; 2 Department of Engineering Science, National Cheng Kung University, No. 1, University Road, Tainan 701, Taiwan; hhlee@mail.ncku.edu.tw

**Keywords:** CMOS image sensor (CIS), layered structures, finite element analysis (FEA)

## Abstract

Image sensors are the core components of computer, communication, and consumer electronic products. Complementary metal oxide semiconductor (CMOS) image sensors have become the mainstay of image-sensing developments, but are prone to leakage current. In this study, we simulate the CMOS image sensor (CIS) film stacking process by finite element analysis. To elucidate the relationship between the leakage current and stack architecture, we compare the simulated and measured leakage currents in the elements. Based on the analysis results, we further improve the performance by optimizing the architecture of the film stacks or changing the thin-film material. The material parameters are then corrected to improve the accuracy of the simulation results. The simulated and experimental results confirm a positive correlation between measured leakage current and stress. This trend is attributed to the structural defects induced by high stress, which generate leakage. Using this relationship, we can change the structure of the thin-film stack to reduce the leakage current and thereby improve the component life and reliability of the CIS components.

## 1. Introduction

Traditional charge-coupled devices (CCDs) have been replaced by CMOS image sensors (CIS), which operate at lower voltage and consume less power than their predecessors. CIS are produced by the CMOS logic process in a system-on-chip (SOC) design environment. CISs are used in consumer electronics, such as digital cameras, optical mice, and mobile phones. However, the CMOS logic process reduces the effectiveness of CIS; a high-element driving current increases the CIS dark current, triggering white pixel noise [1]. Therefore, the performance of the elements, and the adverse effect of leakage currents, must be monitored during the actual process.

By considering thermodynamics, the interfacial stress of the material interface, and its effect on semiconductor films, Cammarata [2] investigated the physical mechanism of surface stress on these films. Spaepe [3] studied athe intrinsic stress at the film’s interface, and Nix and Clement [4] identified the evolution mechanism of the intrinsic tensile stress in the film, namely, crystallite coalescence. Nix and Doerner [5] developed a method for measuring the film stress, described the mechanical properties of film, and modeled the origin of film stress. They also discussed the effect of the film’s microstructure on the deformation process of the film–substrate interface [6].

Wang et al. [7] studied the effect of uniaxial stress on the CIS leakage current by the four-point bending technique (4-PBT). They reported that increasing the tensile stress might further reduce the leakage current of N-type metal-oxide semiconductor (NMOS) image sensors, but renders the leakage current gain at the juncture very sensitive to microstrain. On the other hand, increasing the compressive stress outside the P-type metal-oxide semiconductor (PMOS) increases the leakage current in PMOS image sensors. In other words, increasing the tensile stress enhances the performance of the NMOS image sensor without reducing the leakage current at the juncture. However, for PMOS image sensors under uniaxial compressive stress, the performance of the elements and the effect of leakage current on the elements must be measured.

Rueda [8] also indicated that CIS current flow is reduced under tensile stress, but significantly increased under compressive stress. When the compressive and tensile stresses applied to a CIS are equal, the compressive stress determines the characteristics of the elements. However, as the stress level affects the reverse bias current, the element performance at different leakage currents must be predetermined.

Microminiaturization creates a short channel effect in semiconductors, reducing the critical voltage and increasing the subcritical leakage current. In particular, as the gate oxide layer thins, electrons can more easily penetrate the gate [9,10], generating a gate leakage current. The channel effect can be inhibited by an annular arrangement of the thin films. Although this arrangement effectively reduces the subcritical leakage current, it increases the band-to-band-tunneling (BTBT) leakage current [10,11]. In experiments, a tensile or compressive stress imposed on a semiconductor component affected the electrical performance and the leakage current generated in the off-state of the component [12,13,14].

In general, the relationship between film stress and leakage current significantly affects the characteristics of CIS. Watanabe et al. [15] evaluated the dark current defects of CIS and multiple stress-and-anneal cycles have been found to cause hardening, which was similar to the walk-out in the drain avalanche. Shcherback et al. [16] presented an empirical dark current model for CMOS active pixel sensors. The second part of the model deals with the stress-induced leakage current. The formation of interface traps in these stress regions enhances the generation velocity at the interface and, therefore, causes an increased surface leakage current. In assessing stress, Kim et al. [17] simulated mechanical stress in various backside deep trench isolation (BDTI) structures under a displacement using the FEM. Comparing the final shapes of various BDTIs revealed that it was possible to achieve low-stress structures by decreasing the interfacial curvature between the HfO_X_ and the oxide.

Therefore, the present study discusses the relationship between stress and leakage current in a thin film structure through finite element simulations. We simplified the simulation model (2D structure) to reduce the number of meshes and thereby increase the efficiency of the calculation. We also corrected the material parameters through the single-layer film annealing process experiment and applied the special elements method to simulate the actual stacking sequence process to increase accuracy.

The finite element method not only removes the high cost of equivalent experimental analyses, but also establishes a material properties’ database of stacked materials for specification modification (such as changes in film thickness, stacking order, and stacked materials). The present study provides a computer simulation for semiconductor manufacturers, which can accelerate their technological developments while reducing the experimental cost and increasing the yield of their finished products.

## 2. Analysis Method

### 2.1. Finite Element Analysis

The finite element analysis was performed by ANSYS software (ANSYS, Inc., Canonsburg, PA, USA). The flow was analyzed by a simplified model with set boundary conditions. The material parameters were obtained and the results were simulated.

Since the color filters are stacked in the backend process of the wafer bonding, we first established the material parameters database. At this stage, the different properties of the materials stacked on the wafer, and the different heating and annealing temperatures, impose mechanical structural forces and heat stresses that change the original properties of the elements. By applying finite element analysis, we can accurately observe the stress distributions in the various layers of the material.

**Figure 1:**
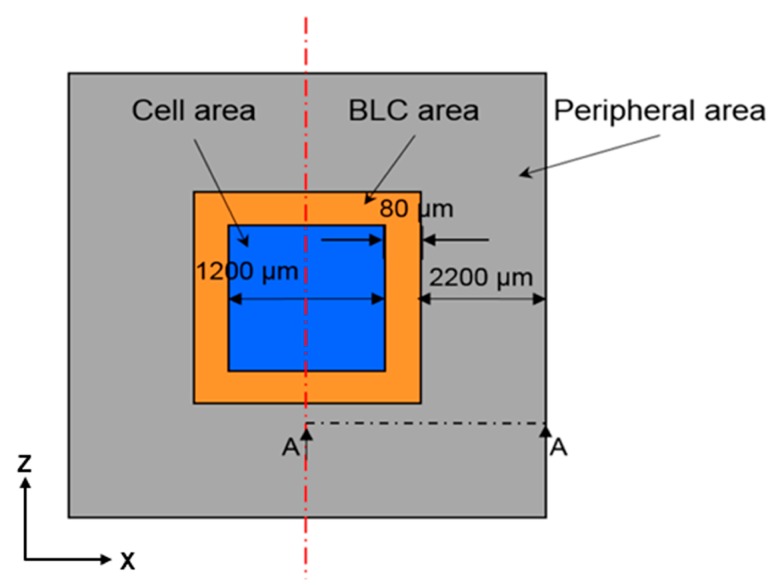
Top view (x-z plane) of the stacked-film architecture. It is a symmetrical structure divided into a cell area, a BLC area, and a peripheral area.

**Figure 2:**
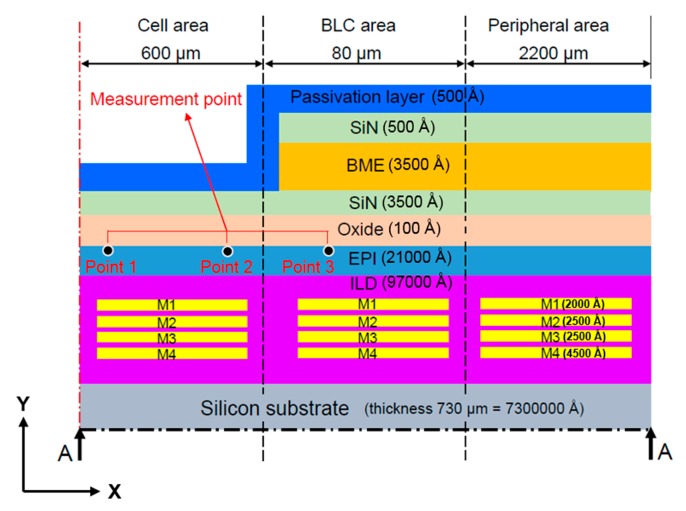
Side view (x-y plane) of Structure A showing a section of the stacked-film architecture along the line A–A.

Figure 1 and Figure 2 are schematics of the film stack architecture in the present study. This architecture was provided by the cooperative manufacturer, Taiwan Semiconductor Manufacturing Company Limited. The light source is received by the cell area in the CIS stack architecture, and obscured by the back-light compensation (BLC) area. The films are annealed, then stacked from bottom to top. As each film is made from a different material, each requires a different annealing temperature. Observations are made at the epitaxial layer, the measuring point in the leakage current measurement area, and the main leakage-current measurement point on the epitaxial layer surface.

As shown in Figure 2, measuring points 1 and 3 are located close to the middle of the cell area (approximately 300 μm from the centerline) and the middle of the BLC area (approximately 640 μm from the centerline, respectively, and measurement point 2 is located near the junction between the cell and BLC areas (approximately 600 μm from the centerline).

#### 2.1.1. Mesh and Convergence

As shown in Figure 1 and Figure 2, because the structure is symmetrical and the model has a defined vertical depth, it can be simplified as a 2D symmetry model. Since the thickness of each layer is very different, we use the thinnest oxide layer as a reference to mesh the element size. In such a 2D plane structure analysis, we use the element “PLANE183” in the software. Lee [18] suggests that, by applying the “PLANE183” element, the model elements can be meshed to quadrilaterals and, subsequently, the simulation results will have greater accuracy.

#### 2.1.2. Boundary Conditions

We set frictionless support on the left side of the model (center axis) to limit the movement of the selected boundary in the x-direction; as the y-direction is free to move, it can be considered a symmetric condition. As the peripheral area structure is continuous and consistent, as shown in Figure 1 and Figure 2, the right side of the model is also set to frictionless support. The temperature load is heated to 350 °C in accordance with the actual manufacturing conditions, then cooled to room temperature.

#### 2.1.3. Simplification of the Simulation

As the film is very thin (thickness = 100 Å at most, versus 7,300,000 Å for the wafer base), the aspect ratio of the finite element model is very large. Thus, the long cell area in the consistent and smooth structure can be shortened by Saint-Venant’s principle, while the film thickness remains unchanged. In this way, the finite element model can be simplified to increase its analytical efficiency.

**Figure 3:**
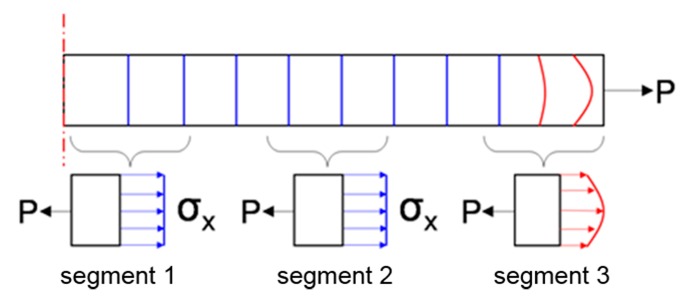
Saint-Venant’s principle showing the stress distribution of a slim member under tension P.

Figure 3 shows the stress distribution of a thin member modeled by Saint-Venant’s principle, The member is stretched by the pulling force P received at its end. Consequently, the transverse cross-section plane near the action location becomes a curved surface (segment 3). However, the cross- section slightly offset from the action spot can be regarded as a plane (segment 2), even under deformation. Since the stress distribution in the cross-section of the member is centrally concentrated near the action location, it is almost uniform away from the action location (segment 1).

#### 2.1.4. Simulating the Thin Film Stack Process

**Figure 4:**
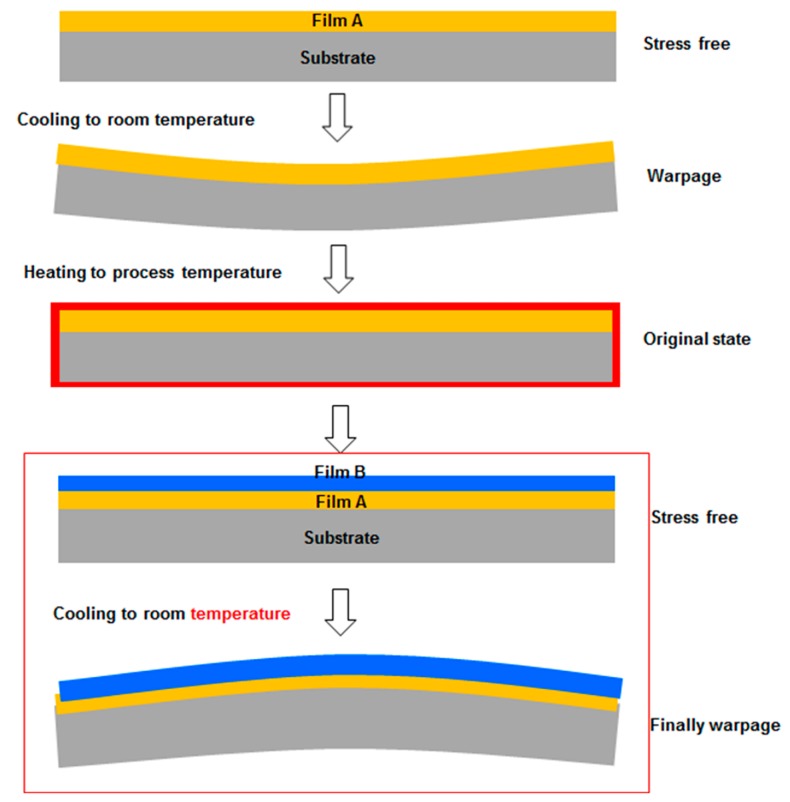
Schematic of the film-stacking process. Each film is annealed before stacking and films are stacked from the bottom up.

As the films are stacked from the bottom up, the film is annealed before stacking. The film stacking process is shown in Figure 4. To accurately simulate the stacking sequence, we apply the special elements method [19].

The special elements method [20] implements the function of an element by modifying the element stiffness. When the first load sub-step of an element in the loading step is dormant or activated, the state is maintained in the overall loading step. Therefore, the dormant elements are invalidated rather than removed.

The stiffness of a dormant element is multiplied by a very small reduction factor. The element load (e.g., pressure and temperature) related to the dormant element is set to zero in the load vector. The mass, damping, strain, and stress stiffening of the dormant element are also set to zero. The dormant elements are not included in the model, but are merely reactivated. When reactivated, the stiffness, mass, damping, and load of the element recover their original values. As the reactivated elements have no strain history, continuous assembly, and annealing can be simulated.

**Figure 5:**
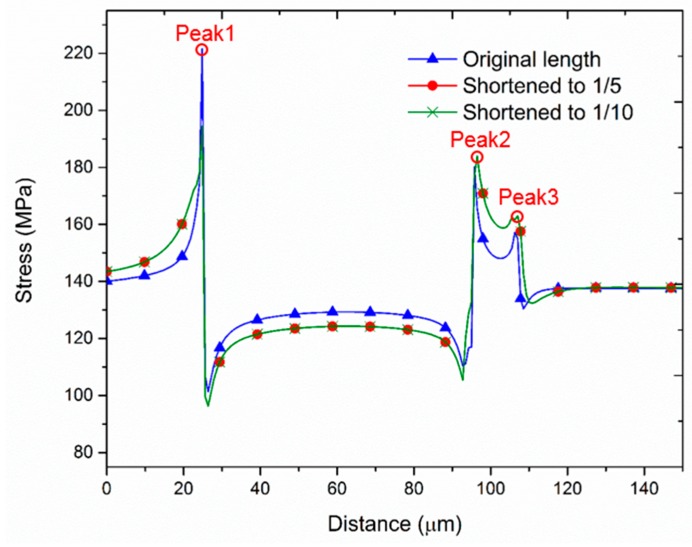
Stress distribution in the BLC area computed by the scaled down model.

Figure 5 shows the simulation results in the epitaxial layer (leakage current measurement layer) of the scaled-down model. It can be observed that the stress trend in the scaled-down model maintains a similar distribution.

Table 1 shows the total number of elements when the model is reduced to 1/10 of the original length. When the element number is reduced, the computation time is likely to be faster. Without compromising stress trends, this method enhances the computational efficiency of the finite element simulation.

#### 2.1.5. Discussion on the Stress Singularity

Elasticity theory is included in the finite element model if there is a geometric structure problem that will cause stress concentration (i.e., the stress is extremely large) at the corresponding position, called the stress singularity point. This is particularly common at the corners.

Hence, we will determine if the peak stress in the simulation results in Figure 5 is a stress singularity point. To do this, we mesh the epitaxial layer elements to a smaller size; if the stress subsequently converges (i.e., the stress becomes smaller), it can be judged as a stress singularity. The results of this evaluation are shown in Table 2: when the epitaxial layer element size was changed from 0.2 μm to 0.05 μm, the peak stress distribution was almost exactly the same. Therefore, we can confirm that these peak stresses are not stress singularity points.

### 2.2. Correction of Material Parameters

**Figure 6:**
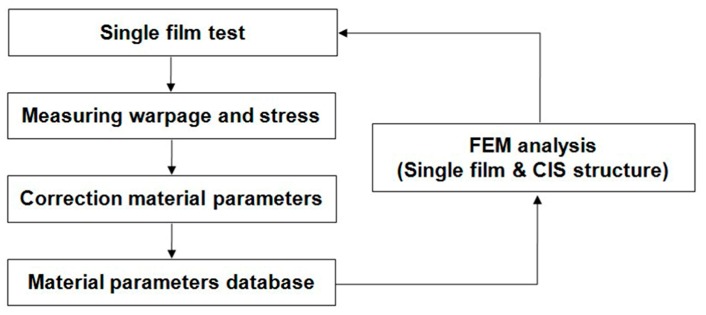
Flowchart for correction of the material parameters.

In the CIS-stack manufacturing process, each material layer is heated and annealed at different temperatures. Therefore, residual stress after the stack process changes the thin-film material properties. To increase the simulation accuracy, the material parameters were corrected by a single-layer stack. In actual manufacturing conditions, a silicon-based wafer is heated, and the film layers are formed at high temperature (165, 220, and 350 °C, from bottom to top), then cooled to room temperature. Next, the wafer is warped and its curvature radius is measured by an ASET SFx100 (KLA-Tencor, Milpitas, CA, USA) measurement instrument. Finally, the film stress is calculated by the Stoney formula [21]. The adjusted parameters of the thin film material parameters shown in Table 3. The Poisson’s ratio, Young’s modulus, and thermal expansion coefficient are corrected by fitting the stress-material parameter correlation curve, and substituting the measured film stress in the curve. Figure 6 is a flow chart of the correction process of the material parameters.

## 3. Simulation Analysis and Experimental Measurements

**Figure 7:**
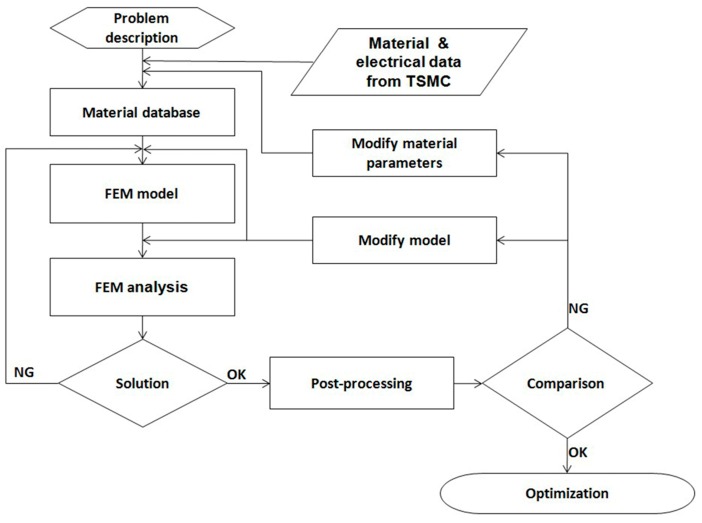
Flowchart of the simulation process.

**Figure 8:**
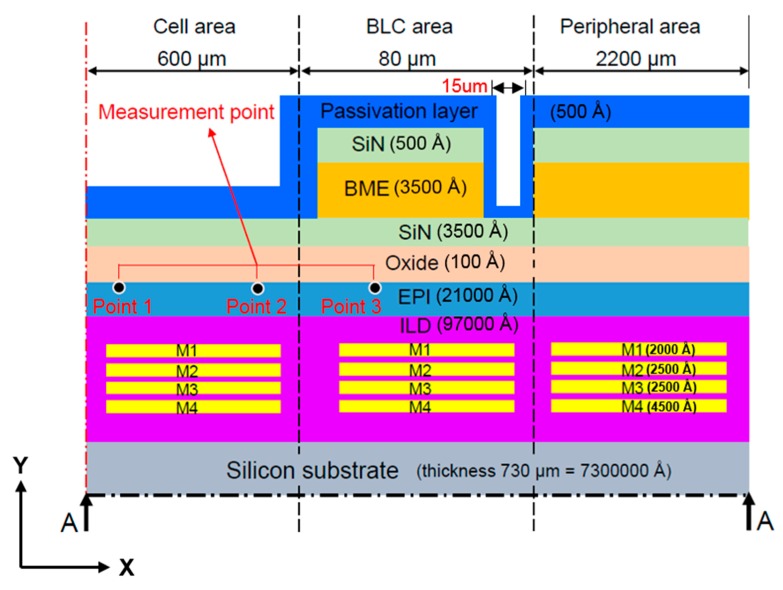
Side view (x-y plane) of Structure B showing the section of the stacked-film architecture along the line A–A.

**Figure 9:**
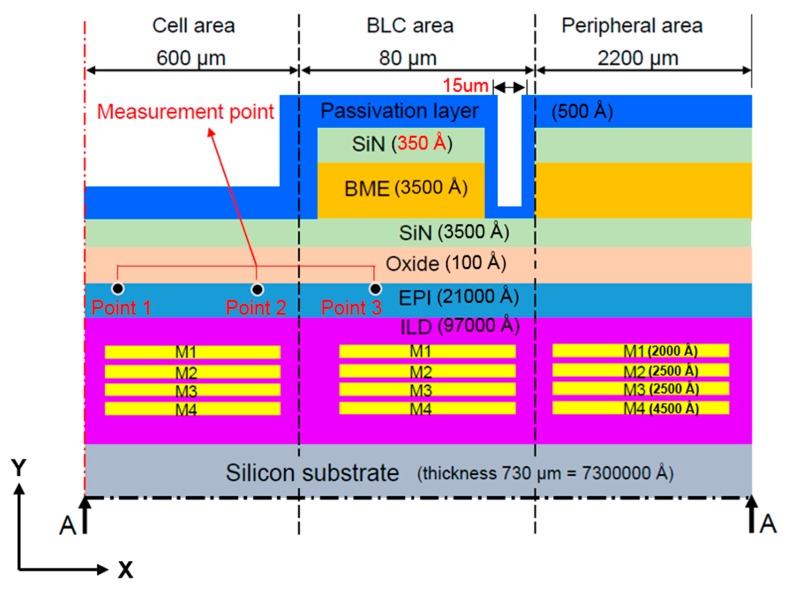
Side view (x-y plane) of Structure C showing the section of the stacked-film architecture along the line A–A.

**Figure 10:**
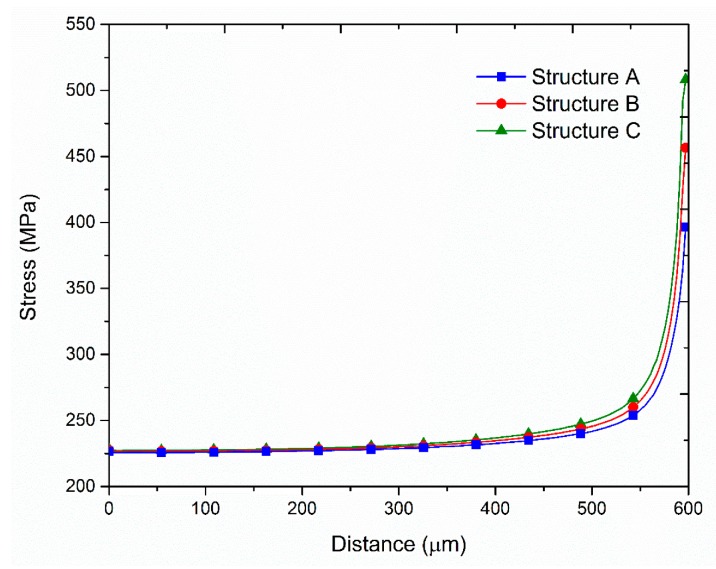
Simulated stress distributions in the cell areas.

**Figure 11:**
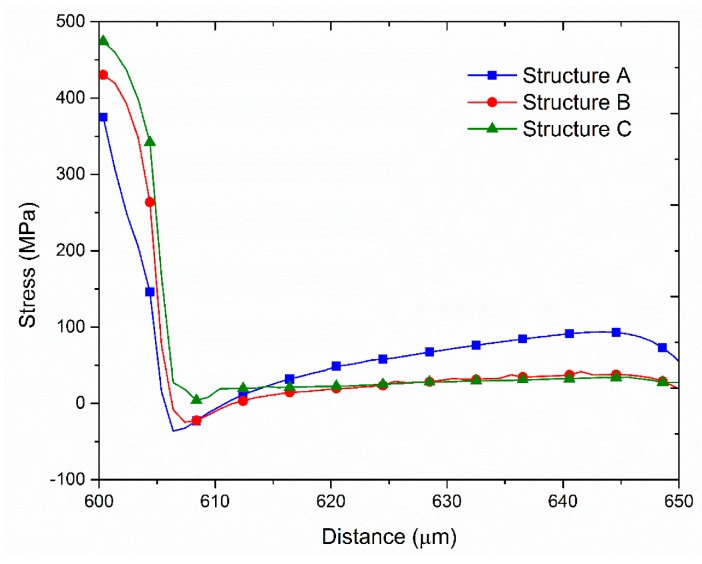
Simulated stress distributions in the BLC areas.

Figure 7 presents the simulation and experimental flow of the optimization. There were three types of film structures: A (Figure 2), B (Figure 8), and C (Figure 9). The difference between these types is that structures B and C have a larger groove (15 μm) in the BLC region compared to structure A; in addition, the thickness of the SiN layer at the top of structure C is decreased from 500 Å (as in structures A and B) to 350 Å. The stress was observed and the leakage current was measured. Figure 10 and Figure 11 show the simulated stress results of the thin-film structures in the cell and BLC areas, respectively. The measured leakage currents, provided by Taiwan Semiconductor Manufacturing Co., Ltd. (Hsinchu, Taiwan), are listed in Table 4.

In both experimental and simulated results, the stress is related to leakage current in the BLC area. Low stress induces a small total leakage current at the three measurement points (Figure 2). In the cell area, as the structure is smooth and slightly changed, the stress distributions are smooth and almost invariant, so the stress–leakage-current relationship cannot be judged.

It can be observed from the simulation results that both the stress and the leakage current of structures B and C are lower than those in structure A, perhaps because the increased groove in the BLC region allows the stress to dissipate. The minor stress differences between structures B and C are due to the different thicknesses of the SiN layers at the top of each structure.

## 4. Results and Discussion

The simulation results were well correlated with the experimental measurements; when the stress was low, the measured leakage current was also low. We now investigate this trend. The simulation performs a static structural analysis, and linear material properties are assumed. The behavior during processing, for example, the concentration of doped semiconductor material and the applied stress, is not considered. The stress distribution in the entire film structure is evaluated after its temperature has decreased to room temperature.

Many studies [22,23,24] have applied 4-PBT or forced wafer bending, in which the electronic characteristics of the elements are altered by an external mechanical stress. However, the applied stress is typically estimated over a wide range. These approaches cannot obtain the actual stress distribution on a small area or a thin layer of a multilayered film on a thick substrate. Therefore, the structure in the present study is based on backside illumination (BSI) technology. We measured the leakage current of the silicon-based transistor at the end rather than at the transistor channel (as in previous studies). Therefore, to explain the correlation between stress and leakage current, we focus on fundamental solid-state quantum theory and the stress-induced defects.

### 4.1. Solid-State Quantum Theory

A solid-state module comprises tens of thousands of atomic systems. During atomic interactions, the original division of the electrons’ energies becomes a banded higher-energy distribution (i.e., an energy band). In the cut-off region of an element, current is still generated by weak reversal development. In addition, the energy of the carriers follows a Boltzmann distribution. The high-energy carriers jump into the conduction band, generating current. Tensile stress increases the interatomic distance and reduces the binding energy of the electrons to their atoms. Consequently, the energy gap is narrowed and the electrons are easily released as free electrons.

Conclusively, stress applied to a pure silicon crystal changes the interatomic distance and the atomic binding force on the electrons. These effects decrease the energy gap in the silicon crystal, and increase the probability of electrons transiting from the valence band to the conduction band at room temperature. On the contrary, when the interatomic distance is shortened under compressive stress, the energy gap increases with the increasing atomic binding force on the electrons, and the electrons are less likely to jump to the conduction band.

**Figure 12:**
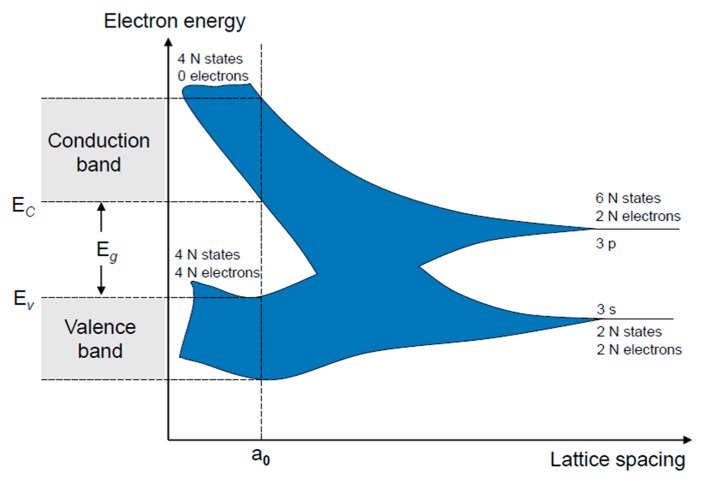
Atomic band structures of silicon [25].

The energy band gap is shown in Figure 12. As the atoms approach each other, the energy levels of the 3*s* and 3*p* states initially split into energy bands. In closer proximity, the 3*s* and 3*p* electrons interact and a mixed band is formed. As the interatomic distance narrows further, the energy band again splits into two energy bands. Finally, at distance a_0_ (defined as the interatomic distance at equilibrium), the upper and lower energy levels are separated by the energy band gap (E*_g_*).

### 4.2. Defects Caused by High Stress

The continued miniaturization of semiconductor device dimensions presents various challenges; for instance, reducing the high gate-pole leakage current induced by a very thin dielectric layer, maintaining high rate of the turn-on and -off currents, and overcoming the short channel effects and high power dissipation.

These problems have been solved by innovative technologies such as strain engineering, shallow junction engineering, low contact resistance, and multilayered interconnections. Strain engineering can improve the channel carrier mobility.

The contact etch-stop layer (CESL) is a popular type of local strained silicon technique. The strained silicon technique, used to cover different materials, causes lattice mismatch. The consequent different stresses in the channel change the component characteristics.

The stress imposed by local strained silicon forms strain channels in a component. This process is exploited in shallow trench isolation, silicidation, CESL, silicon germanium (SiGe), source and drain (S/D) processes, and other related structures.

The CESL above the gate generates stress in the channel, which increases the carrier mobility. However, it also damages the elements to some extent, and defects may occur in the gate oxide and at the silicon/substrate interface.

The effect of CESL on the components was investigated through a series of experiments [12,13,14]. Here, the component size was set to *W*/*L* = 10 μm/10 μm, and the CESL layer conditions were varied as low tensile 380 Å, high tensile 700 Å, and high compressive 700 Å. The silicon on insulator (SOI) thickness was fixed under each CESL condition. The SOI thickness was then varied as 500 Å, 700 Å, and 900 Å, and the *I_D_*–*V_D_* and *I_D_*–*V_G_* diagrams were plotted. To obtain the current and voltage data, we employed a semiconductor parametric analyzer, an inductive–capacitive impedance analyzer and a low leakage current matrix converter. From the curves, we observed the changes in the device characteristics.

**Figure 13:**
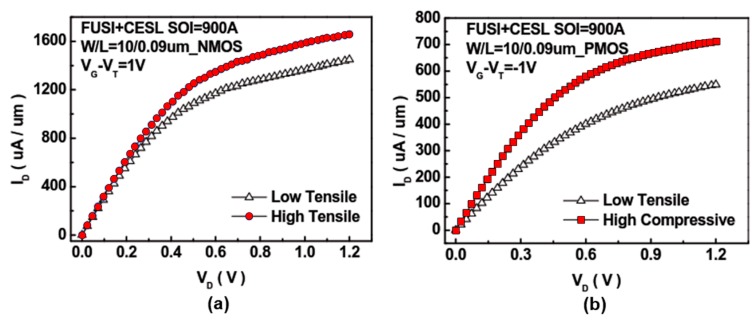
Comparison of *I_D_ - V_D_* diagrams in conducting elements subjected to different layer strains: (**a**) NMOS and (**b**) PMOS [12].

**Figure 14:**
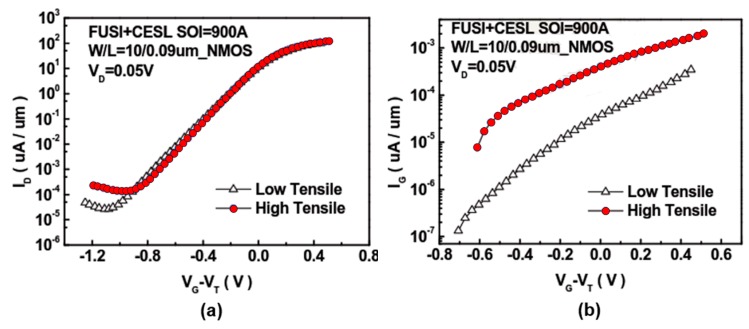
Comparison of *I_D_* and *I_G_* in NMOS non-conducting elements subjected to different layer strains: (**a**) *I_D_*–*V_G_* diagram and (**b**) *I_G_*–*V_G_* diagram [12].

Figure 13 and Figure 14 clarify the effect of strained silicon on the electrical properties of the elements.

Figure 13a shows *I_D_*–*V_D_* diagrams of NMOS. The device with a high tensile force effectively increased the element driving current. The PMOS produced comparable results, as shown in Figure 13b. For NMOS, a slightly higher gate leakage was found in the device with a high tensile force; this is due to the fact that, under high tensile stress, the leakage current increases and defects are generated both in the channel and at the Si/SiO_2_ interface, as shown in Figure 14b.

**Figure 15:**
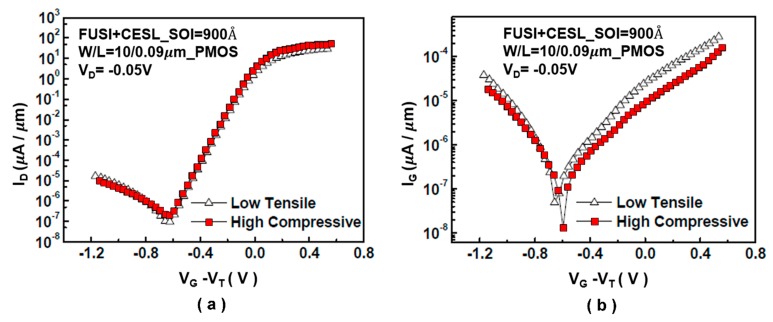
Comparison of *I_D_* and *I_G_* in PMOS non-conducting elements subjected to different layer strains: (**a**) *I_D_*–*V_G_* diagram and (**b**) *I_G_*–*V_G_* diagram [12].

However, in the PMOS component leakage current, the difference in the gate leakage current is not discernible; this is due to the use of a traditional SiN device with a low tensile force in this experiment rather than low compressive force, as shown in Figure 15.

For understanding the defect induced by CESL which was observed in these devices, studies on the densities of oxide-trapped charges and interface traps in transistors have been conducted and reported in [13,14]. These investigations were made by two measurement methods: charge pumping and flicker noise.

**Figure 16:**
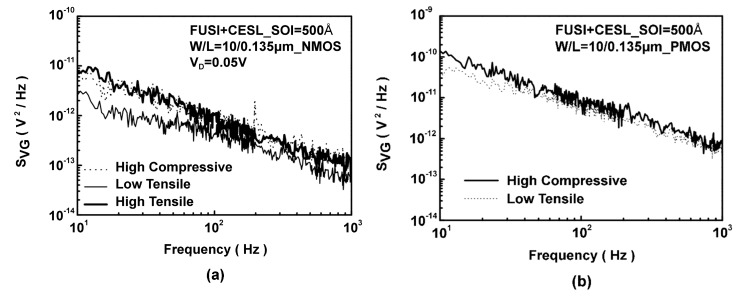
Influence of strain on flicker noise. Comparison of the effects of high- and low-tensile and high-compressive CESL on defects in (**a**) NMOS elements and (**b**) PMOS elements [13].

In this case, low-frequency noise was inspected, as shown in Figure 16. Results indicate higher levels of oxide and interface defects, especially in the high-tensile device with a larger induced stress. Higher oxide and interface defects were found with low-frequency noise, especially in the high compressive PMOS.

**Figure 17:**
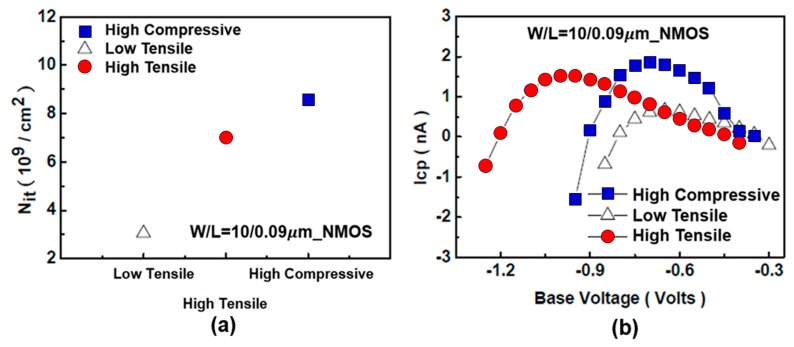
Influence of layer strain on defects in the charge pumping experiment. (**a**) *Icp* currents and (**b**) charge density at the interface (converted from the *Icp* currents), in NMOS devices subjected to different stresses [13].

The charge-pumping measurement was a useful method to confirm the CESL-induced interface defect. For NMOS, a higher nit was observed in higher stress devices (especially in the compression devices), as shown in Figure 17a. This effect was also identified by charge-pumping current (*Icp*) in NMOS, which revealed a higher *Icp*, especially in higher-stress tensile and compressive devices, as shown in Figure 17b. The results confirm that high stress/strain mechanisms cause defects in the elements.

## 5. Verification of the Relation

According to the previous simulation results and the experimental data, both the film structure and the material affect the stress distribution more significantly in the BLC area than in the cell area.

Lowering the stress in the BLC region reduced the measured leakage current; this result is supported by theory and the literature.

Here we verify this trend by simulation and an experiment. The simulation is based on structure C with passivation layer thicknesses of 2*T* and 4*T*, where *T* is the thickness of the original passivation layer 500 Å.

**Figure 18:**
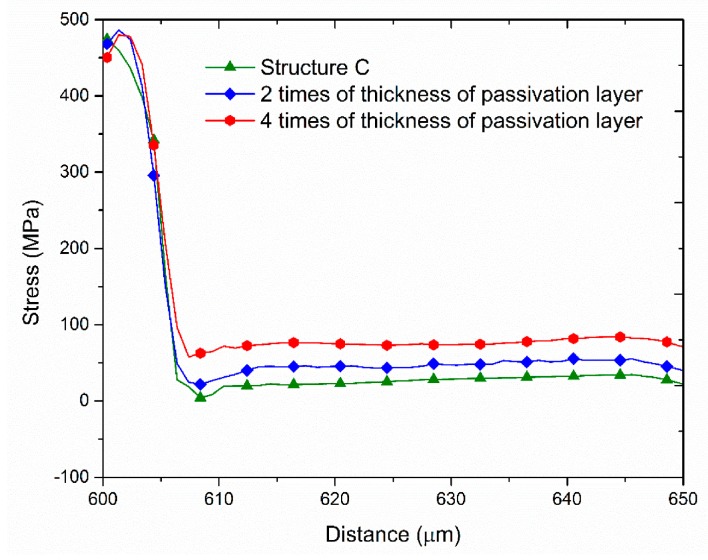
Simulated stress distributions in structures with different passivation layer thicknesses.

Figure 18 shows the simulation result in the BLC area. The stress and the measured leakage current clearly increased with increasing thickness of the passivation layer. Table 5 shows the measured leakage currents in structure C with different passivation layer thicknesses. From the previous results, it shows that the groove can make the stress drop, the leakage current at measurement point 3 has an inverse trend, which may be as the increase in the thickness of the passivation layer but width of the groove (15 μm) remains constant cause the stress around the groove has different changes and measurement point 3 being located closer to the groove. However, from the total leakage point of view, these results are consistent with the previously-observed simulation and experimental trends.

## 6. Conclusions

We applied finite element analysis to the CMOS film-stacking process. The global and local stress distributions in the CIS thin-film structures were simulated during the cooling of the films from high temperature to room temperature. Additionally, the finite element model was simplified to increase the efficiency of the calculation; the material parameters were corrected and the actual stacking sequence process was simulated to increase accuracy.

The simulation results confirmed different stress magnitudes and distributions in film stacks with different architectures. Low stress in the BLC area induces a small leakage current; conversely, high stress causes a high leakage current. As the cell area structure is smooth and robust, the stress distributions are smooth and too similar to discern any relationship between structure and leakage current. To obtain more precise data in the cell area, we must simulate the electro–thermal coupling. The observed trends in the BLC area were related to solid-state quantum theory and the influence of high stress was attributed to defects generated in the stressed materials. Both discussions are consistent.

Simulations of the BLC area or CESL can improve the stack architecture, thus reducing the leakage current and improving the electronic characteristics of the elements. In industrial practice, simulation studies can shorten the technological development time, reduce the experimental cost outlay, and increase the yield of the finished products.

## Figures and Tables

**Table 1:** Number of elements of different model length.

Model Length	Number of Elements
Original	207,605
1/5	108,962
1/10	57,754

**Table 2:** List of peak stress under different element sizes.

Stress	Element Size (μm)			
	0.2	0.15	0.1	0.05
Peak 1	225.2 MPa	225.2 MPa	225.1 MPa	225.1 MPa
Peak 2	182.3 MPa	182.3 MPa	182.3 MPa	182.2 MPa
Peak 3	154.7 MPa	154.7 MPa	154.6 MPa	154.6 MPa

**Table 3:** List of thin film material properties.

Materials	E (GPa)	Poisson Ratio	CTE (ppm/°C)
Oxide	73	0.17	0.55
SiN	130	0.3	10
Epitaxial silicon	190	0.28	2.33
Silicon chip	160	0.28	3
Inter metal dielectric (IMD)	11.7	10.1	1.16

**Table 4:** Leakage current data measured by Taiwan Semiconductor Manufacturing Co., Ltd.

Film Structure	Electrical Data (e^−^/s)		
	Point 1	Point 2	Point 3
Structure A	74.8	9	8.31
Structure B	14.9	8	1.8
Structure C	11.7	10.1	1.16

**Table 5:** Measured leakage current data in Structure C with different passivation layer thicknesses.

Film Structure	Electrical Data (e^−^/s)		
	Point 1	Point 2	Point 3
Structure C	11.7	10.1	1.16
Structure 2*T*	14.6	13	0.9
Structure 4*T*	15.8	18.7	0.84
